# Impact of Anesthetics on Cardioprotection Induced by Pharmacological Preconditioning

**DOI:** 10.3390/jcm8030396

**Published:** 2019-03-21

**Authors:** Sebastian Bunte, Tobias Lill, Maximilian Falk, Martin Stroethoff, Annika Raupach, Alexander Mathes, André Heinen, Markus W. Hollmann, Ragnar Huhn

**Affiliations:** 1 Department of Anesthesiology, University Hospital Duesseldorf, Moorenstr. 5, 40225 Duesseldorf, Germany; Sebastian.Bunte@med.uni-duesseldorf.de (S.B.); Tobias.Lill@uni-duesseldorf.de (T.L.); Maximilian.Falk@uni-duesseldorf.de (M.F.); Annika.Raupach@med.uni-duesseldorf.de (A.R.); Ragnar.Huhn@med.uni-duesseldorf.de (R.H.); 2 Department of Anesthesiology and Intensive Care Medicine, University Hospital Cologne, Kerpener Str. 62, 50937 Cologne, Germany; Alexander.Mathes@uk-koeln.de; 3 Institute of Cardiovascular Physiology, Heinrich-Heine-University Duesseldorf, Universitaetsstr. 1, 40225 Duesseldorf, Germany; Andre.Heinen@uni-duesseldorf.de; 4 Department of Anesthesiology, Amsterdam University Medical Center (AUMC), Location AMC, Meiberdreef 9, 1105 AZ Amsterdam, The Netherlands; M.W.Hollmann@amc.uva.nl

**Keywords:** preconditioning, myocardial infarction, propofol, sevoflurane, dexmedetomidine, milrinone, levosimendan

## Abstract

Anesthetics, especially propofol, are discussed to influence ischemic preconditioning. We investigated whether cardioprotection by milrinone or levosimendan is influenced by the clinically used anesthetics propofol, sevoflurane or dexmedetomidine. Hearts of male Wistar rats were randomised, placed on a Langendorff system and perfused with Krebs–Henseleit buffer (KHB) at a constant pressure of 80 mmHg. All hearts underwent 33 min of global ischemia and 60 min of reperfusion. Three different anesthetic regimens were conducted throughout the experiments: propofol (11 μM), sevoflurane (2.5 Vol%) and dexmedetomidine (1.5 nM). Under each anesthetic regimen, pharmacological preconditioning was induced by administration of milrinone (1 μM) or levosimendan (0.3 μM) 10 min before ischemia. Infarct size was determined by TTC staining. Infarct sizes in control groups were comparable (KHB-Con: 53 ± 9%, Prop-Con: 56 ± 9%, Sevo-Con: 56 ± 8%, Dex-Con: 53 ± 9%; ns). Propofol completely abolished preconditioning by milrinone and levosimendan (Prop-Mil: 52 ± 8%, Prop-Lev: 52 ± 8%; ns versus Prop-Con), while sevoflurane did not (Sevo-Mil: 31 ± 9%, Sevo-Lev: 33 ± 7%; *p* < 0.05 versus Sevo-Con). Under dexmedetomidine, results were inconsistent; levosimendan induced infarct size reduction (Dex-Lev: 36 ± 6%; *p* < 0.05 versus Dex-Con) but not milrinone (Dex-Mil: 51 ± 8%; ns versus Dex-Con). The choice of the anesthetic regimen has an impact on infarct size reduction by pharmacological preconditioning.

## 1. Introduction

Preconditioning interventions, ischemically or pharmacologically, are promising procedures against ischemia and reperfusion injury [1,2,3,4,5,6] that miss clinical routine use because of contradictive clinical data. Over 30 years ago, Murry and colleagues described the phenomenon of ischemic preconditioning; short cycles of non-lethal myocardial ischemia and reperfusion that were able to reduce infarct size significantly before a prolonged ischemic period [5]. A disadvantage of ischemic preconditioning is the massive invasiveness of this intervention, which makes it impracticable for clinical use. Cardioprotection can also be induced by remote preconditioning via tourniquet at the upper or lower limb, for example. It is assumed that humoral factors are released to the blood, which confers myocardial protection. This intervention was shown to induce a strong infarct size reduction [6,7,8]. Because of the lower invasiveness of the intervention compared to direct ischemic preconditioning, effects of remote preconditioning were investigated in clinical trials [9,10]. Unfortunately, the results from large multi-centre trials, e.g., RIPHeart [11] and ERRICA [12], could not show any cardioprotective effect of remote preconditioning in cardiothoracic patients. Propofol, the anesthetic used in these studies, was determined to be an influencing factor for the observed lack of cardioprotection. We previously showed that propofol completely abolished the cardioprotective effect of remote preconditioning [2], but the effect of direct ischemic preconditioning was unaffected [4]. Furthermore, we demonstrated that the time of preconditioning and propofol application is crucial [4].

The cardioprotective effects of ischemic preconditioning can be imitated pharmacologically (e.g., volatile anesthetics, opioids) [13,14,15]. For the phosphodiesterase-3 inhibitor milrinone [1] and the calcium-sensitiser levosimendan [16], infarct size-reducing effects were shown. The infarct size-reducing effects of both drugs were concentration-dependent [3]. Here, we set out to determine the influence of anesthetics on pharmacological preconditioning. We hypothesise that the continuous administration of the different clinically used anesthetics, including propofol, sevoflurane and dexmedetomidine, might have an impact on the preconditioning-induced infarct size reduction of milrinone and levosimendan, respectively.

## 2. Material and Methods

The study was conducted on the baseline of the *Guide for the Care and Use of Laboratory Animals*, published by the National Institutes of Health (Publication number 85–23, revised 1996). The approval of the Animal Ethics Committee of the University of Duesseldorf, Germany has been granted (O27/12).

### 2.1. Surgical Preparation

The surgical preparation was performed as described previously [17]. Male Wistar rats were anesthetised by intraperitoneal injection of pentobarbital (90 mg/kg). Thereafter, animals were thoracotomised for the removal of the hearts. The hearts were mounted on a Langendorff system and perfused with a Krebs–Henseleit buffer (KHB). During the experiment, a constant pressure (80 mmHg) and temperature (37 °C) was maintained [18]. We inserted a 0.1–0.2 mL normal saline-filled balloon into the left ventricle via the left atrium and kept an end-diastolic pressure of 4–7 mmHg. The balloon was coupled to a pressure transducer, which was plugged into an analogue-to-digital converter. The hearts underwent an equilibration period for 20 min. The heart rate, left ventricular end-systolic pressure (LVESP) and left ventricular end-diastolic pressure (LVEDP) were measured continuously and digitised using an analogue-to-digital converter (PowerLab/8SP, ADInstruments Pty Ltd., Castle Hill, Australia) at a sampling rate of 500 Hz. The data were continuously recorded on a personal computer using Chart for Windows v5.0 (ADInstruments Pty Ltd., Castle Hill, Australia). The coronary flow was detected by time-dependent collection of the coronary effluent. The maximal contracture and the time point of maximal contracture were measured in each experiment during ischemia [18].

### 2.2. Experimental Protocol

**Figure 1:**
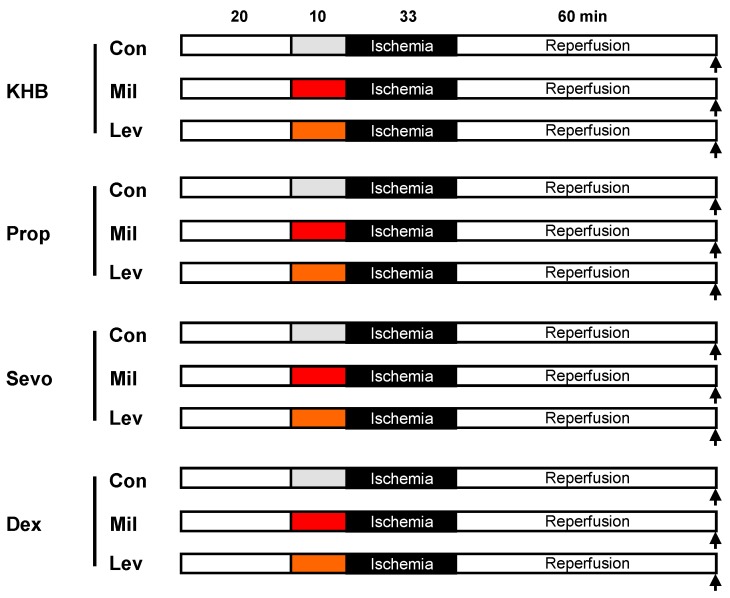
Experimental protocol. KHB, Krebs–Henseleit buffer; Prop, propofol; Sevo, sevoflurane; Dex, dexmedetomidine; Con, control; Mil, milrinone preconditioning (red); Lev, levosimendan preconditioning (orange).

After surgical preparation, all hearts underwent baseline conditions for 20 min and 33 min of global ischemia, followed by 60 min of reperfusion (Figure 1). Global ischemia was achieved by stopping coronary perfusion of the heart with KHB. We randomly assigned the hearts into twelve groups (*n* = 7–8 per group). According to the group, hearts were perfused with either KHB only, propofol (11 µM, Prop), sevoflurane (2.5 Vol.%, Sevo) or dexmedetomidine (1.5 nM, Dex). Propofol and dexmedetomidine were continuously administered via a syringe pump. Sevoflurane was added to the oxygen-nitrogen-gas-mixture via an agent-specific vaporiser and bubbled into the KHB [19]. The sevoflurane concentration was measured using a Datex Ohmeda Capnomac Ultima gas monitor.

Control groups (KHB-Con, Prop-Con, Sevo-Con, Dex-Con): After surgical preparation, rats received KHB or the corresponding anesthetics continuously with no further treatment.

Milrinone (Mil) groups (KHB-Mil, Prop-Mil, Sevo-Mil, Dex-Mil): After surgical preparation, rats received KHB or the corresponding anesthetics continuously and Mil over 10 min before global ischemia. From our own unpublished data, we knew that the lowest cardioprotective concentration of Mil is 1 µM. Therefore, we used this concentration for induction of preconditioning.

Levosimendan (Lev) groups (KHB-Lev, Prop-Lev, Sevo-Lev, Dex-Lev): After surgical preparation, rats received KHB or the corresponding anesthetics continuously and Lev over 10 min before global ischemia. We have previously shown that Lev in a concentration of 0.3 µM is the lowest cardioprotective concentration conferring infarct size reduction [3].

Hearts were dyed with 0.75% triphenyltetrazoliumchloride (TTC) solution at the end of the experiments. The infarct size measurement was carried out using planimetry [18].

### 2.3. Statistical Analysis

Calculation of sample size was done using GraphPad StatMate™ (GraphPad Software, San Diego, CA, USA) and resulted in a group size of *n* = 8, which detected a 25% mean difference and a standard deviation of 11% in infarct size with a power of 80% (α < 0.05 (two-tailed)). Hemodynamic variables were measured continuously and detected during baseline, ischemia and reperfusion. Normality of data was tested with a Kolmogorov–Smirnov Test. To compare hemodynamic variables between groups or between different time points within groups, we used a two-way analysis of variance (ANOVA) and a Tukey post hoc test (GraphPad Software, San Diego, CA, USA). The infarct sizes were determined by an investigator blinded to the experimental groups. A one-way analysis (ANOVA) was chosen, followed by a Tukey post hoc test to analyse infarct size. Statistical analysis of hemodynamic variables and infarct sizes was performed in groups for the respective anesthetic (e.g., all groups with Prop, all groups with Sevo, etc.). Data are presented as mean ± standard deviation (SD). Changes were regarded as statistically significant if *p* < 0.05.

## 3. Results

### 3.1. Animal Characteristics

Table 1 shows body weight, wet and dry heart weight and level and time of maximal ischemic contracture of the experimental groups.

### 3.2. Infarct Size

**Figure 2:**
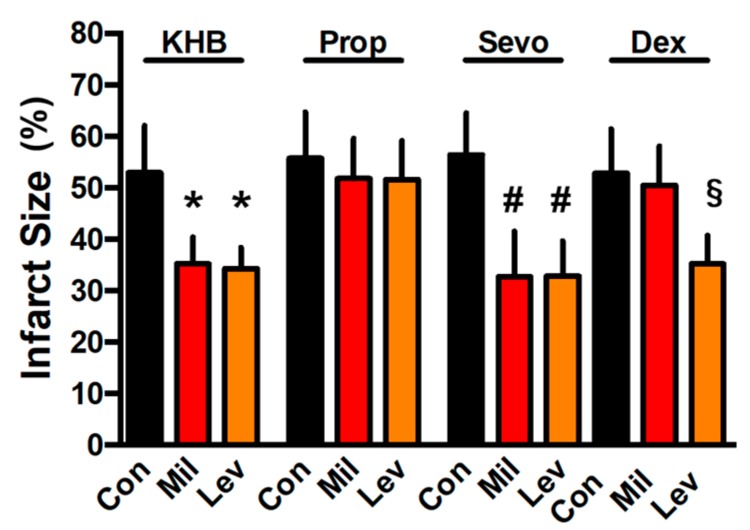
Infarct size measurement. Figure shows the infarct size of controls (Con), preconditioning with milrinone (Mil) and preconditioning with levosimendan (Lev) under different anesthetic regimens. Data are mean ± SD. * *p* < 0.05 versus KHB-Con, ^#^ *p* < 0.05 versus Sevo-Con and ^§^ *p* < 0.05 versus Dex-Con, respectively.

Infarct size in KHB control hearts was 53 ± 9% of the whole heart (Figure 2). Mil and Lev both induced a significant infarct size reduction (KHB-Mil: 35 ± 5%, KHB-Lev: 34 ± 4%; *p* < 0.05 versus KHB-Con). In propofol perfused control hearts, infarct size was 56 ± 9% (Figure 2). Pharmacological preconditioning with Mil or Lev was completely abolished under propofol treatment (Prop-Mil: 52 ± 8%, Prop-Lev: 52 ± 8%; ns versus Prop-Con). In contrast to propofol, Mil and Lev showed significant infarct size reduction under sevoflurane (Sevo-Mil: 31 ± 9%, Sevo-Lev: 33 ± 7%; *p* < 0.05 versus Sevo-Con: 56 ± 8%). Perfusion with dexmedetomidine led to an infarct size of 53 ± 9% in control hearts (Figure 2). Preconditioning with Mil did not induce cardioprotection (Dex-Mil: 51 ± 8%; ns versus Dex-Con). In contrast with Lev-induced preconditioning, infarct size was reduced to 35 ± 5% (*p* < 0.05 versus Dex-Con). There was no difference between control groups of the investigated anesthetic regimens (all *p* > 0.05).

### 3.3. Hemodynamics

Hemodynamic data are shown in Table 2. After ischemia and during reperfusion, LVEDP and coronary flow were statistically different from baseline (Table 2).

## 4. Discussion

The results of the present study show that (1) propofol abolished cardioprotection induced by pharmacological preconditioning with milrinone or levosimendan, (2) sevoflurane had no influence on milrinone- or levosimendan-induced infarct size reduction and (3) dexmedetomidine blocked protection of milrinone, but did not affect levosimendan-induced preconditioning.

Since the disappointing results from clinical trials investigating cardioprotective properties of remote preconditioning came up [11,12,20], the anesthetic regimen, and here especially propofol, was discussed as an influencing factor for the lack of cardioprotection in those studies. In the RIPHeart trial [11], anesthesia was performed 100% with propofol and in the ERRICA trial [12] about 90% of the patients received propofol. Recently, we demonstrated in the rat heart in vivo that propofol completely abolished the infarct size reduction of remote preconditioning, supporting the thesis of propofol being counterproductive in the context of remote preconditioning [2]. In the same study, we showed that the volatile anesthetic sevoflurane did not affect cardioprotection [2]. Direct myocardial ischemic preconditioning under propofol anesthesia was still present [4], but as aforementioned ischemic preconditioning is an extremely invasive and impractical intervention that is not suitable for clinical routine, with the exception of cardiac surgery or cardiac catheterisation. Fortunately, the effect of ischemic preconditioning can be mimicked pharmacologically, e.g., with milrinone or levosimendan [1,3,16]. The calcium sensitiser levosimendan and the phosphodiesterase-3 inhibitor milrinone are drugs indicated for treatment of acute heart failure [21,22]. Both drugs are routinely used in the operating room or the intensive care unit, so the cardioprotective properties of these substances are of interest just like possible negative influencing factors, e.g., anesthetics.

For milrinone and levosimendan, in each case we used the lowest concentration to induce the strongest cardioprotective effect. Recently, we demonstrated that 0.3 µM levosimendan is the lowest concentration that conferred the strongest infarct size reduction [3]. We know from own unpublished data that for milrinone, 1 µM is the concentration that leads to pronounced cardioprotective effects. Higher concentrations of milrinone could not maximise the infarct size reduction.

Propofol abolished the cardioprotective effect of both pharmacological preconditioning interventions, whereas sevoflurane had no impact on levosimendan- or milrinone-induced preconditioning. These results support the hypothesis of propofol being a negative influencing factor on conditioning strategies. Kottenberg et al. demonstrated that remote preconditioning under anesthesia with isoflurane decreased troponin levels in cardiac surgical patients after cardiopulmonary bypass, but this effect was abrogated under propofol anesthesia [23]. When solely administered, there was no difference in troponin levels between isoflurane and propofol. One has to distinguish between a continuous administration and a preconditioning stimulus of the volatile anesthetic sevoflurane. Preconditioning can be induced through three cycles of intermittent administration of sevoflurane alternated with phases of reperfusion before ischemia. Previously, we showed that continuous administration of sevoflurane for the whole study period (baseline, ischemia and reperfusion) had no effect on infarct size in the rat heart in vivo [2,4]. In a recent study, we demonstrated that intermittent sevoflurane administration (preconditioning stimulus) reduced infarct size significantly, whereas the continuous application of sevoflurane had no effect on infarct size [24]. In line with our experimental findings, Bein et al. [25] reported that in coronary surgery, patients that received a continuous administration of 1 MAC sevoflurane from induction to start of cardiopulmonary bypass did not result in any additional protection, compared with the control group. However, when the administration of sevoflurane before cardiopulmonary bypass was interrupted for 10 min, an improved myocardial performance and decreased postoperative troponin T were observed. These data suggest that the interrupted administration is crucial for clinically relevant cardioprotective effects. Cardioprotective strategies can be influenced by confounding factors, such as aging or diabetes. We previously showed that as a variable, aging abolished the infarct size reduction of ischemic and pharmacological preconditioning [26,27,28,29]. Co-morbidities, such as diabetes [30], or medications, such as beta-blockers [30], impact cardioprotection. By using young and healthy rat hearts, all possible confounding factors were excluded in the present study to investigate exclusively the effect of various anesthetics on pharmacological preconditioning.

However, the results from levosimendan- and milrinone-induced preconditioning under dexmedetomidine administration are inconsistent. While the cardioprotective effect of levosimendan was unaffected, milrinone-induced preconditioning was completely abolished. A clinical interaction and mutual influence between milrinone and dexmedetomidine is described [31]. We assume that this interaction is responsible for the loss of the milrinone effect under dexmedetomidine. However, we were not aware of this interaction when we initiated the present study, and an elucidation of this drug interaction was beyond the scope of the study. To the best of our knowledge, there is no study addressing the underlying mechanism and/or interaction of both substances in more detail. We can only speculate that signaling pathways induced by both dexmedetomidine and milrinone might interfere with each other. One possible candidate pathway is the p38MAPK/ERK signaling cascade, which is a well-described underlying mechanism in preconditioning interventions. Yeda et al. [32] showed that dexmedetomidine abolished the ischemia/reperfusion (I/R) injury-induced increase in p38 MAPK in diabetic rats, whereas Sanada et al. [33] demonstrated activation of p38 MAPK by milrinone-induced preconditioning in dogs. Thus, it is conceivable that combining both drugs might exert a neutralising effect on each other. Furthermore, levosimendan is 10–30 times more potent an inotropic agent than milrinone, albeit with a lower myocardial oxygen consumption [34]. Thus, levosimendan exerts a positive inotropic effect without disturbing the energy balance of the heart, which could be an advantage during I/R injury.

Furthermore, we did not investigate whether the sole application of milrinone with dexmedetomidine administration before and after the preconditioning stimulus would lead to different results. However, this approach would not represent the clinical scenario.

The present data have a purely descriptive manner and it is a limitation of the study that we did not establish the underlying blockade mechanism, which was beyond the scope of our study. The aim of our study was to elucidate anesthetic drugs as a possible confounding factor for conditioning strategies.

## 5. Conclusions

The results of our current study show that the choice of the anesthetic had an impact on the cardioprotective effect of pharmacological preconditioning. While protection was completely blocked by propofol, it was unaffected by the use of sevoflurane and inconsistent with dexmedetomidine. Thus, the anesthetic regimen might be an important influencing factor that should be considered when cardioprotective agents are used.

## Figures and Tables

**Table 1:** Weights and ischemic contracture.

		*n*	Body Weight (g)	Heart Weight Dry (g)	Heart Weight Wet (g)	Time of Max. Ischemic Contracture (min)	Level of Max. Ischemic Contracture (min)
KHB	Con	8	301 ± 10	0.14 ± 0.02	1.47 ± 0.12	15:46 ± 1:42	54 ± 7
	Mil	7	299 ± 23	0.15 ± 0.01	1.46 ± 0.11	16:27 ± 1:18	54 ± 11
	Lev	7	291 ± 21	0.14 ± 0.01	1.32 ± 0.14	15:39 ± 0:39	56 ± 12
Prop	Con	8	286 ± 17	0.13 ± 0.02	1.44 ± 0.11	16:17 ± 1:05	59 ± 12
	Mil	8	303 ± 23	0.16 ± 0.02	1.46 ± 0.08	16:00 ± 1:10	55 ± 4
	Lev	8	299 ± 26	0.15 ± 0.01	1.47 ± 0.11	16:26 ± 1:59	61 ± 11
Sevo	Con	7	300 ± 08	0.12 ± 0.01	1.34 ± 0.06	14:26 ± 0:23	82 ± 13
	Mil	7	298 ± 19	0.13 ± 0.01	1.30 ± 0.06	15:05 ± 1:53	70 ± 13
	Lev	7	297 ± 11	0.13 ± 0.01	1.29 ± 0.07	14:40 ± 1:07	81 ± 11
Dex	Con	8	293 ± 18	0.14 ± 0.03	1.42 ± 0.09	15:21 ± 2:10	67 ± 17
	Mil	8	290 ± 19	0.14 ± 0.02	1.41 ± 0.07	16:16 ± 1:54	55 ± 9
	Lev	7	296 ± 11	0.15 ± 0.02	1.42 ± 0.09	16:14 ± 1:23	55 ± 10

Data are mean ± standard deviation (SD). KHB, Krebs–Henseleit buffer; Prop, propofol; Sevo, sevoflurane; Dex, dexmedetomidine; Con, control; Mil, milrinone; Lev, levosimendan.

**Table 2:** Hemodynamic variables.

Group		Baseline	PC	Reperfusion	
				30	60
*Heart Rate (bpm)*					
KHB	Con	333 ± 40	314 ± 42	279 ± 92	275 ± 62
	Mil	327 ± 48	347 ± 44	242 ± 61	275 ± 43
	Lev	318 ± 39	312 ± 33	274 ± 34	258 ± 36
Prop	Con	308 ± 32	294 ± 32	183 ± 69 *	180 ± 67 *
	Mil	313 ± 30	293 ± 27	237 ± 46	212 ± 54
	Lev	316 ± 50	300 ± 51	208 ± 77	212 ± 74
Sevo	Con	296 ± 23	271 ± 28	246 ± 20	215 ± 45
	Mil	345 ± 38	367 ± 32	221 ± 60	266 ± 63
	Lev	304 ± 37	300 ± 27	271 ± 37	253 ± 32
Dex	Con	308 ± 27	286 ± 15	248 ± 53	247 ± 28
	Mil	318 ± 43	309 ± 39	257 ± 46	264 ± 26
	Lev	331 ± 54	328 ± 36	304 ± 36	265 ± 54
*Phasic LVP (mmHg)*					
KHB	Con	117 ± 16	122 ± 10	17 ± 8 *	22 ± 7 *
	Mil	130 ± 16	115 ± 13	15 ± 5 *	19 ± 6 *
	Lev	109 ± 17	116 ± 17	25 ± 15 *	25 ± 11 *
Prop	Con	132 ± 20	126 ± 20	17 ± 8 *	28 ± 14 *
	Mil	118 ± 18	119 ± 19	20 ± 4 *	31 ± 11 *
	Lev	130 ± 12	131 ± 13	20 ± 9 *	27 ± 6 *
Sevo	Con	144 ± 17	145 ± 23	25 ± 14 *	32 ± 10 *
	Mil	129 ± 19	143 ± 24	26 ± 13 *	39 ± 18 *
	Lev	136 ± 24	139 ± 11	27 ± 12 *	35 ± 10 *
Dex	Con	124 ± 16	113 ± 20	21 ± 10 *	29 ± 12 *
	Mil	123 ± 15	125 ± 17	27 ± 13 *	30 ± 8 *
	Lev	126 ± 11	127 ± 10	24 ± 14 *	24 ± 10 *
*CF (mL·min^−1^)*					
KHB	Con	17 ± 3	15 ± 4	10 ± 1 *	8 ± 1 *
	Mil	18 ± 2	16 ± 2	9 ± 2 *	8 ± 2 *
	Lev	15 ± 2	17 ± 2	7 ± 2 *	5 ± 2 *
Prop	Con	17 ± 1	16 ± 1	9 ± 1 *	7 ± 1 *
	Mil	16 ± 2	18 ± 3	10 ± 1 *	8 ± 2 *
	Lev	16 ± 1	19 ± 2	10 ± 2 *	8 ± 2 *
Sevo	Con	15 ± 3	14 ± 3 *	9 ± 2 *	7 ± 2 *
	Mil	17 ± 3	18 ± 3 *	11 ± 4 *	9 ± 3 *
	Lev	14 ± 3	17 ± 2	8 ± 3 *	7 ± 3 *
Dex	Con	16 ± 3	14 ± 4	8 ± 1 *	6 ± 1 *
	Mil	16 ± 2	14 ± 2	7 ± 3 *	7 ± 1 *
	Lev	19 ± 2	18 ± 3	9 ± 2 *	7 ± 2 *

Data are mean ± SD. KHB, Krebs–Henseleit buffer; Prop, propofol; Sevo, sevoflurane; Dex, dexmedetomidine; Con, control; Mil, milrinone; Lev, levosimendan. * *p* < 0.05 versus baseline.
